# Cofactor Binding Protects Flavodoxin against Oxidative Stress

**DOI:** 10.1371/journal.pone.0041363

**Published:** 2012-07-19

**Authors:** Simon Lindhoud, Willy A. M. van den Berg, Robert H. H. van den Heuvel, Albert J. R. Heck, Carlo P. M. van Mierlo, Willem J. H. van Berkel

**Affiliations:** 1 Laboratory of Biochemistry, Wageningen University, Wageningen, The Netherlands; 2 Biomolecular Mass Spectrometry and Proteomics, Bijvoet Center for Biomolecular Research and Utrecht Institute for Pharmaceutical Sciences, Utrecht University, Utrecht, The Netherlands; Uni. of South Florida, United States of America

## Abstract

In organisms, various protective mechanisms against oxidative damaging of proteins exist. Here, we show that cofactor binding is among these mechanisms, because flavin mononucleotide (FMN) protects *Azotobacter vinelandii* flavodoxin against hydrogen peroxide-induced oxidation. We identify an oxidation sensitive cysteine residue in a functionally important loop close to the cofactor, i.e., Cys69. Oxidative stress causes dimerization of apoflavodoxin (i.e., flavodoxin without cofactor), and leads to consecutive formation of sulfinate and sulfonate states of Cys69. Use of 7-chloro-4-nitrobenzo-2-oxa-1,3-diazole (NBD-Cl) reveals that Cys69 modification to a sulfenic acid is a transient intermediate during oxidation. Dithiothreitol converts sulfenic acid and disulfide into thiols, whereas the sulfinate and sulfonate forms of Cys69 are irreversible with respect to this reagent. A variable fraction of Cys69 in freshly isolated flavodoxin is in the sulfenic acid state, but neither oxidation to sulfinic and sulfonic acid nor formation of intermolecular disulfides is observed under oxidising conditions. Furthermore, flavodoxin does not react appreciably with NBD-Cl. Besides its primary role as redox-active moiety, binding of flavin leads to considerably improved stability against protein unfolding and to strong protection against irreversible oxidation and other covalent thiol modifications. Thus, cofactors can protect proteins against oxidation and modification.

## Introduction

Proteins are sensitive to oxidative damage and the resulting protein modifications often have considerable biological effects. For example, protein oxidation has been suggested as a causative or contributory factor in many diseases [1]. Furthermore, oxidized proteins have been found to increase in aged organisms, suggesting that protein oxidation contributes to aging [2], [3]. Of particular interest is oxidation of methionine and cysteine residues, because this conversion happens for a wide variety of proteins and often affects their biological activity [4], [5].

Oxidation of methionine to its sulfoxide state can be stimulated by biological oxidants such as H_2_O_2_, but also by environmental oxidants like ozone [6]. Methionine oxidation can be (partially) reversed by the action of methionine sulfoxide reductases [7]. Such repair of oxidative damage suggests a potential for *in vivo* regulation of protein function by reversible methionine oxidation [8], [9], [10], [11].

**Figure 1:**
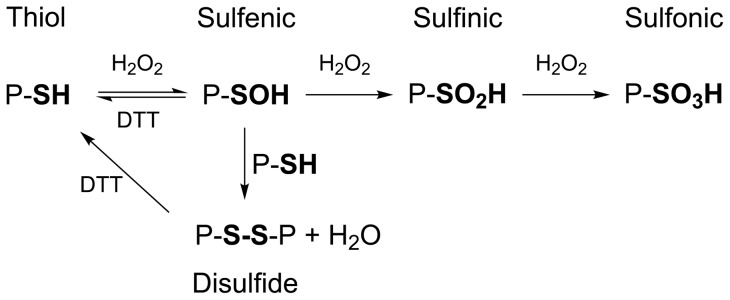
Oxidation states of protein cysteines, and their reversibility by DTT. Schematic diagram showing steps involved in hydrogen peroxide-induced oxidation and DTT-induced reduction of protein cysteines.

The most oxidation sensitive and reactive amino-acid residue is cysteine. The presence of reactive cysteine residues is indicative of functionality, and affects many biochemical processes [12]. Oxidation of protein thiols involves consecutive formation of sulfenic, sulfinic and sulfonic acids [13], as shown in Figure 1. Sulfenic acids are intermediates during formation of disulfide bonds [14], but have also been recognized as naturally occurring states of cysteinyl residues critical to catalytic and regulatory processes [15], [16]. Recent evidence suggests that protein sulfenic acids are widespread posttranslational oxidative modifications in healthy mammalian tissues, and that their presence increases in response to elevated concentrations of H_2_O_2_ [17], [18], [19], [20], [21].

When protective mechanisms fail, protein thiols may be further oxidized to the sulfinic acid state or the irreversible sulfonic acid state [22], [23]. Consequently, protein sulfinylation and sulfonylation has been linked to cell aging and cell death [24]. Aging has been investigated for several purified proteins. For example, in case of *p*-hydroxybenzoate hydroxylase from *Pseudomonas fluorescens*, oxidative aging produces sulfonic acid derivatives of Cys116 [22]. Substitution of this cysteine by a serine precludes oxidation and prevents protein aggregation [25]. In case of *Trigonopsis variabilis* D-amino acid oxidase, stress-induced oxidation results in decreased protein stability due to the irreversible formation of the sulfinic acid state of Cys108 [26]. However, in case of peroxidoredoxin, the sulfinic acid state of the active site cysteine can be rapidly transformed into the catalytically active thiol state [27]. This reduction probably requires specific enzymes. In contrast, matrix metalloproteinase-7 is activated by oxidation of Cys70 into sulfinic acid [28].

In this study, we report the sensitivity towards oxidative damage of a 179-residue flavodoxin from *A. vinelandii*. The protein functions as an FMN-dependent one-electron transporter to nitrogenase in the nitrogen fixation pathway of this bacterium [29]. Flavodoxin adopts the α–β parallel topology [30], also referred to as the doubly-wound or flavodoxin-like topology, which is one of the most common folds observed. Flavodoxins are the structurally most investigated flavoproteins and have emerged as prototypes for the investigation of protein folding [31], [32], [33], [34], [35], [36], [37]. Furthermore, flavodoxins are found in many prokaryotes, but also as protein domains in eukaryotes (e.g., in nitric oxide synthase) [38].

**Figure 2:**
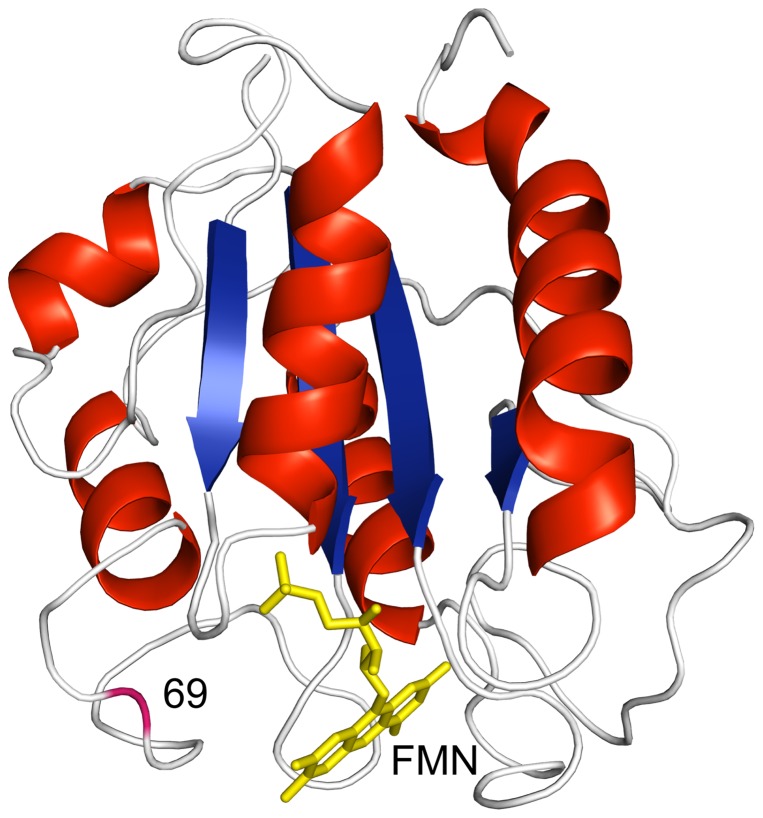
Cartoon drawing of the X-ray structure of flavodoxin from *A. vinelandii*. α-Helices are shown in red, β-strands in blue and loops in white. The FMN cofactor is coloured yellow and the backbone of residue 69 is coloured magenta. Note that this residue is in immediate vicinity of FMN. The X-ray structure is of the C69A variant of the protein (pdb ID 1YOB) [30], in which the single cysteine at position 69 is replaced by alanine. This protein variant is largely similar to flavodoxin regarding both redox potential of holoprotein and stability of apoprotein [42], [54].

*A. vinelandii* flavodoxin contains two residues that are potentially sensitive to oxidation: a methionine at position 30 and a cysteine at position 69. Whereas the side chain of the methionine is hidden in the protein interior, the cysteine is located in a loop at the surface and in immediate vicinity of the FMN cofactor (Figure 2), as is the case for many other flavodoxins. NMR studies on the nearly identical flavodoxin from *Azotobacter chroococcum* show that this loop is involved in intermolecular interactions with the nitrogenase enzyme system [39]. Indeed, biological activity is lost upon covalent dimerization of *A. vinelandii* flavodoxin molecules through formation of an intermolecular disulfide bridge. [40], [41], [42]. Currently, the exact physiological role of Cys69 is unclear. It is suggested to function as an additional redox active centre to FMN, or to be involved in an activation/deactivation mechanism of the protein [43].

Upon removal of the non-covalently bound flavin from flavodoxin, apoflavodoxin is generated. NMR data show that apoflavodoxin strongly resembles flavodoxin, except for dynamic disorder in the flavin-binding region [44]. Due to this flexibility, disulfide-linked dimerization of apoflavodoxin happens more rapidly than is the case for flavodoxin [40]. Here, we use H_2_O_2_ to induce oxidative stress on flavodoxin as well as apoflavodoxin. We address the effects of oxidative stress through analysis of the various protein modifications formed, and highlight the importance of the cofactor in protecting flavoproteins against irreversible oxidation and potentially other covalent thiol modifications.

## Results

### Cys69 of (apo)flavodoxin is sensitive to oxidation

**Figure 3:**
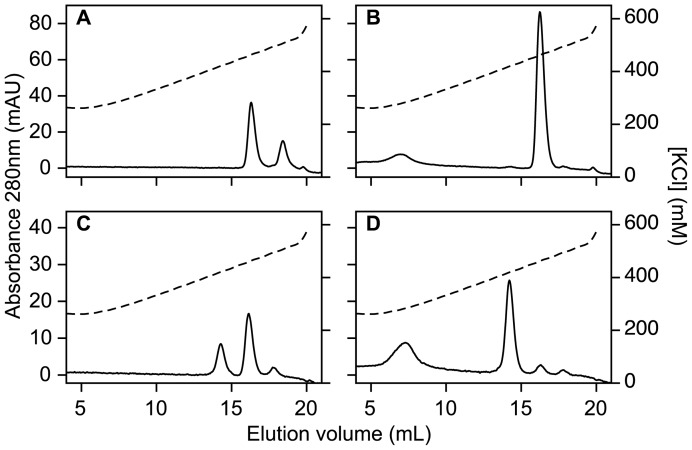
MonoQ anion exchange chromatography elution profiles of flavodoxin and apoflavodoxin. (A) Flavodoxin (40 μM). (B) Flavodoxin (40 μM), kept in the presence of 10 mM DTT for a period of 10 min. (C) Apoflavodoxin (50 μM). (D) Apoflavodoxin (50 μM), kept in the presence of 10 mM DTT for a period of 10 min. Gradient composition: buffer A is 25 mM Tris-HCl pH 8.0, and buffer B is 25 mM Tris-HCl pH 8.0, containing 1 M KCl. Flow rate is 1.0 mL/min. Dashed lines show conductivities of elution buffers. The molecule eluting at 7 mL is DTT. Temperature is 25°C.

MonoQ anion exchange chromatography of freshly purified flavodoxin shows the presence of two protein species, eluting at 470 and 510 mM KCl, respectively (Figure 3a). Depending on protein preparation, the first eluting species amounts to about 50–70% of total protein. Incubation of flavodoxin with 10 mM DTT for a period of 10 min causes complete conversion of the second into the first eluting species (Figure 3b). This observation shows that Cys69 is reversibly modified in the second eluting species. Preparations of Cys69Ala (C69A) flavodoxin elute as a single species during anion exchange chromatography.

During size-exclusion chromatography, flavodoxin elutes almost exclusively as monomer. Thus, the second flavodoxin species detected by anion exchange chromatography is not disulfide linked protein dimer, but instead involves another thiol modification. We propose that the species eluting at 510 mM KCl at pH 8 (Figure 3a), is protein with Cys69 in the sulfenic acid state (Figure 1), because it is converted into protein that elutes at 470 mM KCl after incubation with DTT (Figure 3b). This DTT-induced conversion is not expected to happen when flavodoxin's thiol would be oxidised to the sulfinic or sulfonic acid state [22], [23], [45]. In addition, because the p*K*_a_ of sulfenic acid is about 5.9 [16], at pH 8 one expects retarded elution on an anion exchange column of the sulfenic acid state of flavodoxin compared to non-oxidised protein, just as we observe (Figure 3a).

In case of apoflavodoxin, MonoQ anion exchange chromatography reveals two major protein species, eluting at 420 and 460 mM KCl, respectively (Figure 3c). The fraction of the second species increases proportional to the time during which apoprotein stock is left in absence of DTT. Subsequent incubation of apoflavodoxin with 10 mM DTT causes almost complete conversion of this second species into the first eluting one (Figure 3d). Thus, Cys69 is reversibly modified in the second eluting species. Size-exclusion chromatography shows that freshly prepared apoflavodoxin is predominantly monomeric but slowly dimerizes during storage (data not shown). Apoflavodoxin dimers elute at 460 mM KCl in MonoQ anion exchange chromatography. These dimers are disulfide-linked, because upon incubation with excess DTT, they convert into monomeric species.

### Reactivity with DTNB and NBD-Cl

Incubation of apoflavodoxin (stored in presence of DTT prior to experiments, to avoid protein dimerization) as well as of flavodoxin with DTNB leads to formation of nearly stoichiometric amounts of TNB. In case of flavodoxin, protein modification proceeds rather slowly and takes about 30 min to complete, whereas apoflavodoxin is modified within 2 min (for experimental circumstances see Materials and Methods). The observed difference in reactivity shows that Cys69 is better accessible to Ellman's reagent in apoprotein than in holoprotein, consistent with the observation that the flavin-binding region of apoflavodoxin (and thus of the loop in which Cys69 resides) is flexible, whereas this region is rigid in flavodoxin [44]. To explain stoichiometric formation of TNB in case of flavodoxin, holoprotein with Cys69 in the sulfenic acid state must react with DTNB. Indeed, reactivity of sulfenic acids with TNB has been reported [46].

**Figure 4:**
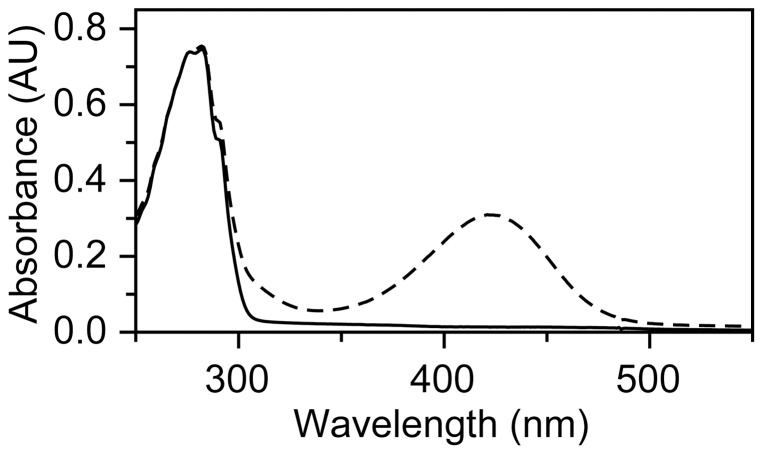
Spectroscopic characteristics of apoflavodoxin modified with NBD. Apoprotein (20 μM) before (solid line) and after (dashed line) incubation with 200 μM NBD-Cl for 1 hour. Modified protein absorbs maximally at 420 nm, which is characteristic for the presence of a thiol-NBD conjugate [47].

**Figure 5:**
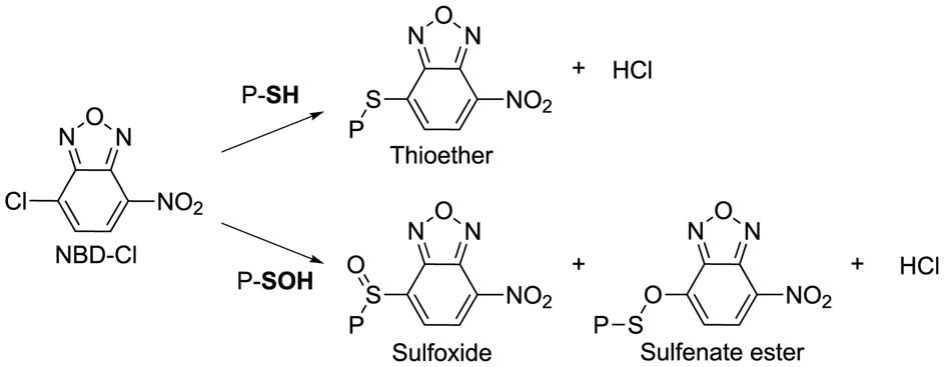
Reactions of NBD-Cl with protein cysteine and sulfenic acid states. Schematic diagram showing steps involved in reaction of NBD-Cl with cysteine and sulfenic acid.

Flavodoxin does not react appreciably with NBD-Cl, whereas the reaction of this reagent with freshly prepared, DTT-pre-treated, apoflavodoxin takes 60 min to complete. The absorption maximum at 420 nm of the apoflavodoxin adduct formed (Figure 4) is characteristic for thioether linked NBD (Figure 5) [47]. Hence, Cys69 of apoflavodoxin stock is in the thiol state. No sulfoxide adduct is detected, because (i) apoflavodoxin preparation involves TCA precipitation of holoprotein and at the resulting low pH sulfenic acid is unstable [15], and (ii) apoflavodoxin is stored in presence of 10 mM DTT prior to NBD-Cl treatment, causing sulfenic acid to transform into thiol.

### Native MS and LC-MS analysis

**Figure 6:**
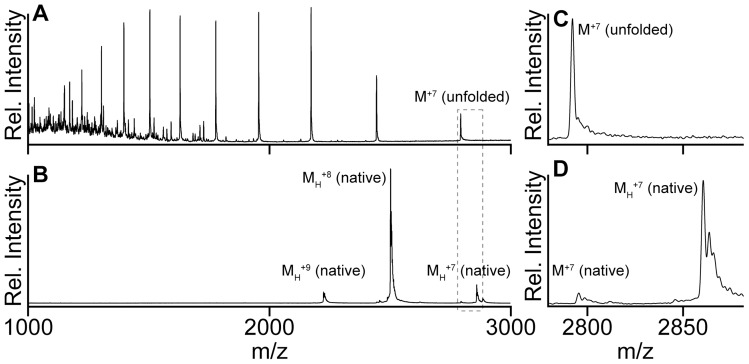
LC-MS and native MS spectra of flavodoxin. (A) LC-MS spectrum of flavodoxin. (B) Nano-electrospray mass spectrum of flavodoxin. M_H_^+n^ represents flavodoxin monomer with n positive charges, and M^+n^ is apoflavodoxin monomer with n positive charges. (C and D) Spectra of area indicated by the grey contour in (A) and (B), respectively. Flavodoxin (5 µM) is in 50 mM ammonium acetate, 0.1 mM EDTA, pH 6.8.

By using native mass spectrometry [48] we detect flavodoxin, which has an MS spectrum that shows a narrow charge state distribution (i.e., +7 to +9) (Figure 6). The corresponding molecular mass equals (19995±3) Da, which closely agrees with the expected mass of the holoprotein with cysteine in the thiol state (Table 1). Freshly prepared, DTT-pre-treated, native apoflavodoxin exhibits a similar narrow distribution of charge states (i.e., +7 to +9), and the corresponding molecular mass is (19538±3) Da (Table 1). The mass difference between holo- and apoprotein establishes that flavodoxin indeed contains a single, non-covalently bound, FMN cofactor.

**Table 1:** Native mass spectrometry of (apo)flavodoxin.

Species	Label in Figure 6	Expected mass (Da)	Measured mass (Da)	Δ(mass) (Da)
Apoflavodoxin	M	19531.8	19538±3	+6.2 Da
Flavodoxin	M_H_	19987.1	19995±3	+7.9 Da

Monomeric protein species are labelled M, Molecular masses of FMN is 455.3 Da. Expected mass and measured mass are average masses.

During LC-MS analysis, we first inject flavodoxin into the LC part of the set-up and subsequently electrospray it into a mass spectrometer. In the reverse-phase chromatography step, which involves use of acetonitrile and TFA, flavodoxin loses FMN and subsequently apoprotein unfolds. As a consequence of this unfolding, the corresponding LC-MS spectrum displays a broad distribution of charged protein molecules (i.e., +7 to +17) (Figure 6), clearly distinct from the native MS data. Again due to unfolding, the LC-MS spectrum of apoflavodoxin is identical to the one of holoprotein. Unfolded apo- and holoprotein both are observed with a molecular mass of (19533±1.5) Da, which agrees very well with their predicted masses (i.e., 19531.8 Da; Table 2), assuming that cysteine is in the thiol state and the co-factor has been released from the holoprotein. In case of holo- as well as apoprotein we observe no sulfenic acid, because acetonitrile-TFA usage during the reversed phase chromatography step of LC-MS and resulting low pH causes instability of sulfenic acid [15].

**Table 2:** LC-MS analysis of (apo)flavodoxin.

Species	Label in Figure 8	Expected mass (Da)	Measured mass (Da)	Δ(mass) (Da)
Apoflavodoxin	M	19531.8	19533±1.5	+1.2 Da
Apoflavodoxin +1 O	MO	19547.8	Not observed	-
Apoflavodoxin +2 O	MO2	19563.8	19565±2	+1.2 Da
Apoflavodoxin +3 O	MO3	19579.8	19582±2	+2.2 Da
Apoflavodoxin + NBD	M-NBD	19695.9	19697±2	+1.1 Da
Apoflavodoxin +1 O + NBD	MO-NBD	19711.9	19713±2	+1.1 Da
Apoflavodoxin dimer	D	39063.6	39066±5	+2.4 Da

Monomeric protein species are labelled M. Molecular masses of NBD-Cl and NBD are 199.6 and 164.1 Da, respectively. Expected mass and measured mass are average masses.

The above observations show that flavodoxin and apoflavodoxin are sensitive to thiol oxidation, prompting further investigation of this phenomenon.

### Hydrogen peroxide-mediated oxidation of apoflavodoxin

**Figure 7:**
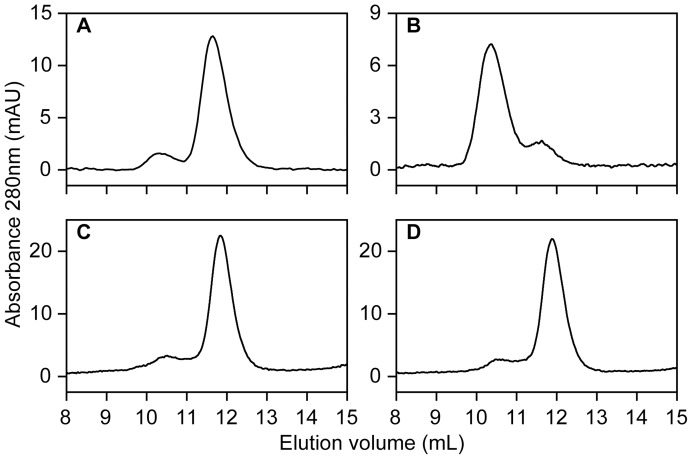
Superdex 75 size exclusion chromatography elution profiles of apoflavodoxin. (A) Apoflavodoxin (38 μM). (B) Apoflavodoxin (38 μM) after 22 h incubation with 10 mM H_2_O_2_ at 4°C. (C) C69A apoflavodoxin (79 μM). (D) C69A apoflavodoxin (79 μM) after 24 h incubation with 10 mM H_2_O_2_ at 4°C. Flow rate is 0.5 mL/min and temperature is 25°C.

To prevent intermolecular disulfide bond formation in apoflavodoxin, protein is stored in presence of 10 mM DTT. Just before incubation with H_2_O_2_, this reducing agent is removed to avoid interferences with H_2_O_2_. Size-exclusion chromatography shows that freshly prepared apoflavodoxin is predominantly monomeric (Figure 7A). Upon incubation of apoflavodoxin with 10 mM H_2_O_2_ the relative amount of protein dimers strongly increases (Figure 7B). In contrast, upon incubation of C69A apoflavodoxin with H_2_O_2_ no protein dimerization takes place (Figure 7C–D).

**Figure 8:**
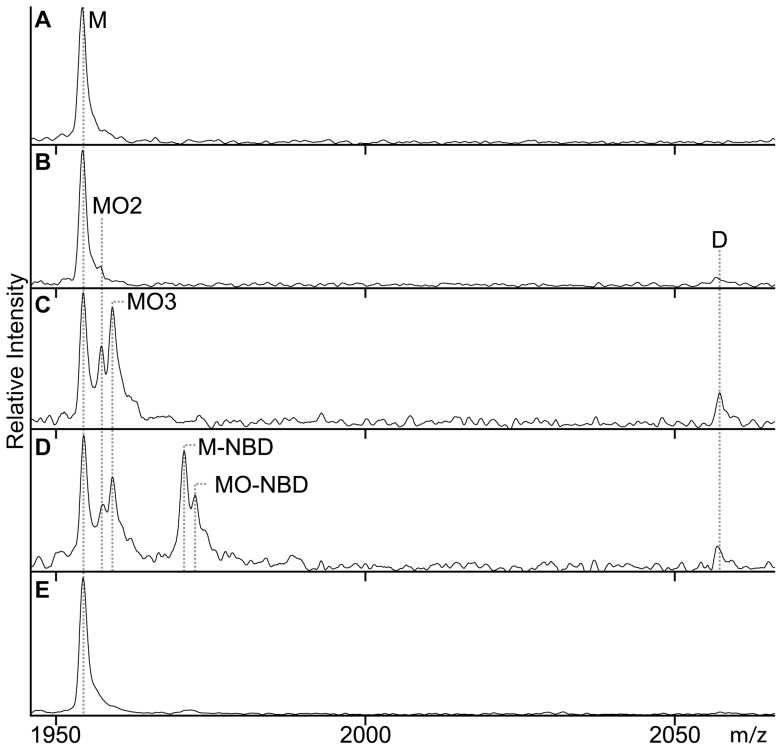
Monitoring of (apo)flavodoxin under H_2_O_2_-induced oxidative stress by LC-MS. For clarity, the zoomed in LC-MS regions that display the +10 charge state of monomer and the +19 charge state of dimer are shown (i.e., m/z range of 1940 to 2070); however, analysis is done on the whole m/z range. (A) Apoflavodoxin. (B) Apoflavodoxin incubated for 30 min with 10 mM H_2_O_2_. (C) Apoflavodoxin incubated for 30 min with 100 mM H_2_O_2_. (D) Apoflavodoxin incubated for 30 min with 200 µM NBD-Cl and 100 mM H_2_O_2_. (E) Flavodoxin incubated for 30 min with 190 µM NBD-Cl and 100 mM H_2_O_2_. Protein concentration is 5 µM and incubations were done at room temperature. M represents apoflavodoxin monomer with non-oxidised thiol; MO, MO2 and MO3 are the sulfenic, sulfinic and sulfonic acid states of apoflavodoxin, respectively. M-NBD is monomer protein with the thiol adduct of NBD-Cl, MO-NBD is monomer protein with the sulfenic acid adduct of NBD-Cl, and D represents disulfide-linked apoflavodoxin dimer.

We used LC-MS to follow H_2_O_2_-induced oxidation of apoflavodoxin at room temperature. Figure 8a shows the LC-MS spectrum of apoprotein prior to its incubation with H_2_O_2_. Clearly, only monomeric, non-oxidised, apoflavodoxin is detected. After incubating apoflavodoxin with 10 mM H_2_O_2_ for 30 min, the corresponding LC-MS spectrum shows appearance of signal of protein dimer (measured mass (39066±5) Da) (Figure 8B). The population of dimer rises upon increasing incubation time. Again, this dimer is disulfide-linked, because upon incubation with 10 mM DTT it converts into monomeric species. Besides a tiny population of protein in sulfinic acid state (labelled MO2 in Fig 6b), no other oxidation products of monomeric apoflavodoxin are detected, even when incubation with H_2_O_2_ lasts up to two hours. Thus, under the conditions applied, Met30 of apoflavodoxin is not susceptible to peroxide-mediated sulfoxidation.

Incubation of apoflavodoxin with 100 mM H_2_O_2_ for a period of 30 min leads to detection of four protein species by LC-MS (Figure 8C). These species are: non-oxidised protein monomer (M), protein dimer, and protein monomer in sulfinic or sulfonic acid state (labelled MO2 and MO3, respectively). Upon incubation with DTT, only the apoflavodoxin dimer disappears from the LC-MS spectrum, showing that oxidation of Cys69 to its sulfinic or sulfonic acid state is irreversible with respect to this reducing agent. No ion intensity at the expected mass of the sulfenic acid state of apoflavodoxin is observed (Figure 8C). Again, usage of acetonitrile and TFA during the reversed phase chromatography step destabilizes sulfenic acid, preventing its detection by LC-MS. Upon increasing the H_2_O_2_-incubation period, dimer intensity in LC-MS spectra no further raises. The latter phenomenon is in line with Figure 1, which shows the proposed mechanism for apoflavodoxin oxidation. The most likely first event during H_2_O_2_ treatment is formation of sulfenic acid [16]. Subsequently, this intermediate either reacts rapidly with Cys69 of another apoflavodoxin monomer to form protein dimer or oxidises further under influence of H_2_O_2_ to sulfinic and sulfonic acid states of apoflavodoxin. Upon increasing the time of incubation with 100 mM H_2_O_2_ beyond 30 min, competition between both processes depletes sulfenic acid intermediate and hence no increase in relative population of apoflavodoxin dimer is detected.

### Trapping of sulfenic acid state during apoflavodoxin oxidation

To support actual formation of a sulfenic acid intermediate during oxidation of apoflavodoxin, H_2_O_2_-mediated oxidation of apoprotein was studied in presence of NBD-Cl. This procedure enables spectroscopic and mass discrimination between NBD adducts of thiols and sulfenic acids [46], [47], [49]. Now, a 30 min incubation at room temperature gives rise to formation of only a minor population of protein dimers (Figure 8D) that does not increase upon prolonged incubation. Besides dimer, LC-MS detects five additional monomeric apoflavodoxin species, including protein non-oxidised thiol (M) and protein in sulfinic (MO2) or sulfonic (MO3) acid state. In addition, protein with the thiol adduct of NBD-Cl (labelled M-NBD) and with the sulfenic acid adduct of NBD-Cl (labelled MO-NBD) are identified (Figure 4). This observation shows that the sulfenic acid intermediate can be trapped by NBD-Cl and indeed forms during H_2_O_2_-induced oxidation of apoflavodoxin.

### Hydrogen peroxide-mediated oxidation of flavodoxin

In contrast to apoflavodoxin, incubation of flavodoxin for a 30 min period with 100 mM H_2_O_2_ and 190 µM NBD-Cl does not cause formation of protein dimer, nor of protein species MO2, MO3, M-NBD and MO-NBD, as LC-MS reveals (Figure 8E). Clearly, flavodoxin is much better protected against formation of these products than apoflavodoxin. In addition, like in apoflavodoxin, Met30 of flavodoxin is not susceptible to peroxide-mediated sulfoxidation.

Does H_2_O_2_-mediated oxidation of flavodoxin cause production of holoprotein with Cys69 in the sulfenic acid state? Whereas the LC-MS methodology prevents detection of protein with sulfenic acid, due to usage of acetonitrile-TFA during reversed phase chromatography, MonoQ anion exchange chromatography can detect such species, as discussed (Figure 3A, B). Using the latter methodology, we indeed observe that upon incubation of flavodoxin with 10 mM H_2_O_2_ for a period of 30 min at room temperature, approximately 85% of protein is in the sulfenic acid state (i.e., elutes at 510 mM KCl, data not shown). However, in holoprotein, neither subsequent further oxidation to sulfinic and sulfonic acid nor formation of intermolecular disulfides is observed (Figure 8).

## Discussion

### Cofactor binding protects flavodoxin against oxidative stress

Many proteins require binding of a cofactor to be functional. However, the role of such ligands in protein oxidation and stability is scarcely addressed [50]. Binding of cofactors stabilizes proteins against global unfolding [51]. Due to bound FMN, the stability against global unfolding of flavodoxin is much higher than that of apoflavodoxin. As a result, global unfolding of flavodoxin is a rare event, occurring approximately once every 3 hours [33]. The stability of flavodoxin is so high that FMN needs to be released first before global unfolding of the protein can occur [33]. Hydrogen/deuterium exchange studies revealed that FMN binding protects the majority of flavodoxin's residues against local protein unfolding [44], [52], [53]. In contrast to apoflavodoxin, the backbone amide protons of several residues in the flavin-binding region of flavodoxin exchange extremely slowly with deuterons of deuterium oxide [44], [52]. This slow exchange reflects the rigidity of the flavin-binding region in flavodoxin, which is caused by the many interactions that exist between FMN and apoprotein. These observations highlight that a secondary role flavin binding has is protecting flavoproteins against unfolding.

The study presented here highlights another secondary role of flavin binding: i.e., the importance of the FMN cofactor in protecting flavodoxin against irreversible oxidative damage. The data show that apoflavodoxin is much more susceptible to H_2_O_2_-induced oxidative stress than holoprotein. This is likely caused by an increased accessibility of Cys69. Furthermore, dissociation of FMN potentially affects the p*K*a of this residue. Both apo- and holoprotein first reversibly oxidise to their sulfenic acid states (Figure 1). Subsequently, in case of apoflavodoxin, sulfenic acid is irreversibly oxidised to sulfinic and sulfonic acid states. In addition, part of the sulfenic acid population reacts with unmodified apoflavodoxin monomers, thereby generating disulfide-linked protein dimers. In contrast, in case of flavodoxin, oxidation beyond the sulfenic acid state is not detected. Flavodoxin does not dimerize and is also considerably less susceptible to covalent modification by TNB and NBD-Cl than apoflavodoxin. Apparently, upon sulfenylation, the side chain of Cys69 becomes even further stabilized and protected against subsequent modification.

In conclusion, besides its primary role as redox-active moiety, binding of FMN leads to considerably improved stability of flavodoxin against unfolding and to strong protection against oxidative stress.

## Materials and Methods

### Chemicals

FMN and 1,4-dithiothreitol (DTT) were from Sigma-Aldrich. Ammonium acetate and 5,5′-dithiobis-(2-nitrobenzoic acid) (DTNB) were from Merck, and H_2_O_2_ (30% w/v) was from Fisher. NBD-Cl from Acros Organics was dissolved in dimethyl sulfoxide.

### Protein purification and preparation of apoprotein

Wild-type flavodoxin II from *A. vinelandii* strain ATCC 478 was expressed in *E. coli* TG2 (pAV34) and purified as described [54], with omission of DTT. The purified protein migrated as one homogeneous band in SDS-polyacrylamide gel electrophoresis. Holoprotein gave rise to an A276/A450 absorbance ratio of 5.4. C69A flavodoxin was prepared as described [54]. To produce apoflavodoxin, FMN was removed from flavodoxin by trichloroacetic acid (TCA) precipitation [54]. Before dissolving in 300 mM Tris-HCl pH 8.5, protein pellet was washed twice with 3% (w/v) TCA containing 1 mM DTT [31]. To avoid disulfide bond formation between apoprotein monomers, apoflavodoxin was stored in the presence of 10 mM DTT. Prior to experiments, by using Biogel-P6DG gel filtration (BioRad), flavodoxin as well as apoflavodoxin were brought into 50 mM ammonium acetate, 0.1 mM EDTA, pH 6.8. All experiments with holo- and apoprotein were performed using this buffer.

### Protein modification

Reactions of flavodoxin (41 μM) and apoflavodoxin (31 μM) with DTNB were carried out at 20°C according to the method of Ellman [55] with the modifications of Habeeb [56]. DTNB concentration was 200 μM. Time-dependent release of 2-nitro-5-thio-benzoate anion (TNB) was measured at 412 nm (ε_412_(TNB)  = 13.6 mM^−1^cm^−1^). Apoflavodoxin (20 μM) was also treated for 1 h at 20°C with 200 μM NBD-Cl (stock contained 100 mM NBD-Cl in dimethyl sulfoxide). In another experiment, apoflavodoxin was first oxidised at room temperature with 10 mM H_2_O_2_ for periods of 10 and 90 min, respectively, and subsequently incubated with NBD-Cl, as described. In addition, DTT-pre-treated flavodoxin (28 μM) was incubated for 100 min at 20°C with 190 μM NBD-Cl.

### Fast protein liquid chromatography

Size-exclusion chromatography was performed using a Superdex 75 HR 10/30 column. Anion exchange chromatography was done using a MonoQ HR5/5 column. All FPLC separations were performed at room temperature using an ÄKTA Explorer (GE healthcare).

### Spectral analysis

Absorption spectra were recorded at 25°C on a Hewlett Packard 8453 diode array spectrophotometer. Flavodoxin's molar absorption coefficient of 10.8 mM^−1^cm^−1^ at 450 nm was determined by recording absorption spectra of protein in presence and absence of 0.1% (w/v) SDS, using a molar absorption coefficient for free FMN of 12.2 mM^−1^cm^−1^ at 445 nm.

### Nano-electrospray mass spectrometry

Native MS spectra of protein were obtained by using an LC-T nano-electrospray ionization orthogonal time-of-flight mass spectrometer (Micromass, Manchester, UK), operating in positive ion mode. Spraying conditions were: needle voltage 1250–1450 V, cone voltage 50–125 V and source temperature 80°C. To prepare nano-electrospray needles, borosilicate glass capillaries (Kwik-FilTM, World Precision Instruments Inc., Sarasota, FL) and a P-97 puller (Sutter Instrument Co., Novato, CA) were used. The resulting needles were subsequently coated with a thin gold layer (∼500 Å) by using an Edwards Scancoat six Pirani 501 sputter coater (Edwards High Vacuum International, Crawley, UK).

### Liquid chromatography-mass spectrometry (LC-MS)

For liquid chromatography use was made of a LC-10AD Shimadzu two pump system coupled to a SPD-10A UV-VIS Shimadzu detector, which was set to 210 nm. Protein samples were brought onto a Vydac 218TP54 C18-reversed phase analytical column (250×4.6 mm internal diameter, 5 µm particle size) and a Vydac 218TP5115 C18-reversed phase microbore column (150×1 mm internal diameter, 5 µm particle size), which was protected by an Optimize technologies OPTI-GUARD C18-reversed phase 1 mm guard column. Flow rate of the mobile phase through the analytical Vydac column was 0.6 mL/min. Flow rate through the microbore column was 0.05 mL/min. The mobile phase was a mixture of solvent A (milli-Q water containing 0.05% (v/v) trifluoroacetic acid (TFA)) and solvent B (95% (v/v) acetonitrile, containing 0.05 (v/v) TFA). This mixture initially contained 40% solvent B for a period of 5 min. Subsequently, in a time span of 10 min, concentration of solvent B was increased to 60% and kept isocratically for 5 min. After this time period, solvent mixture was brought back in 5 min to initial composition (i.e., 40% B). The LC-T mass spectrometer of the LC-MS set-up operated in positive ion mode with 3 kV capillary voltage, 50 V cone voltage, 120°C source temperature and 250 L/h desolvation gas flow.
